# Supplementary material for: Replication cycle timing determines phage sensitivity to a cytidine deaminase toxin/antitoxin bacterial defense system
Source: PLoS Pathog. 2023 Sep 8;19(9):e1011195. doi: 10.1371/journal.ppat.1011195 (PMC10511110; doi:10.1371/journal.ppat.1011195)
Supplement: S1 Fig — Shown are the raw images of Northern blots of avcI RNA and Western blots of AvcD-6xHis during rifampicin treatment, spectinomycin treatment, T5 infection, and T7 infection. Gray triangle corresponds to avcI transcript (~280 nt) and black triangle corresponds to AvcD-6xHis (60 kDa). (DOCX) [file ppat.1011195.s001.docx]

**Supplemental**

**
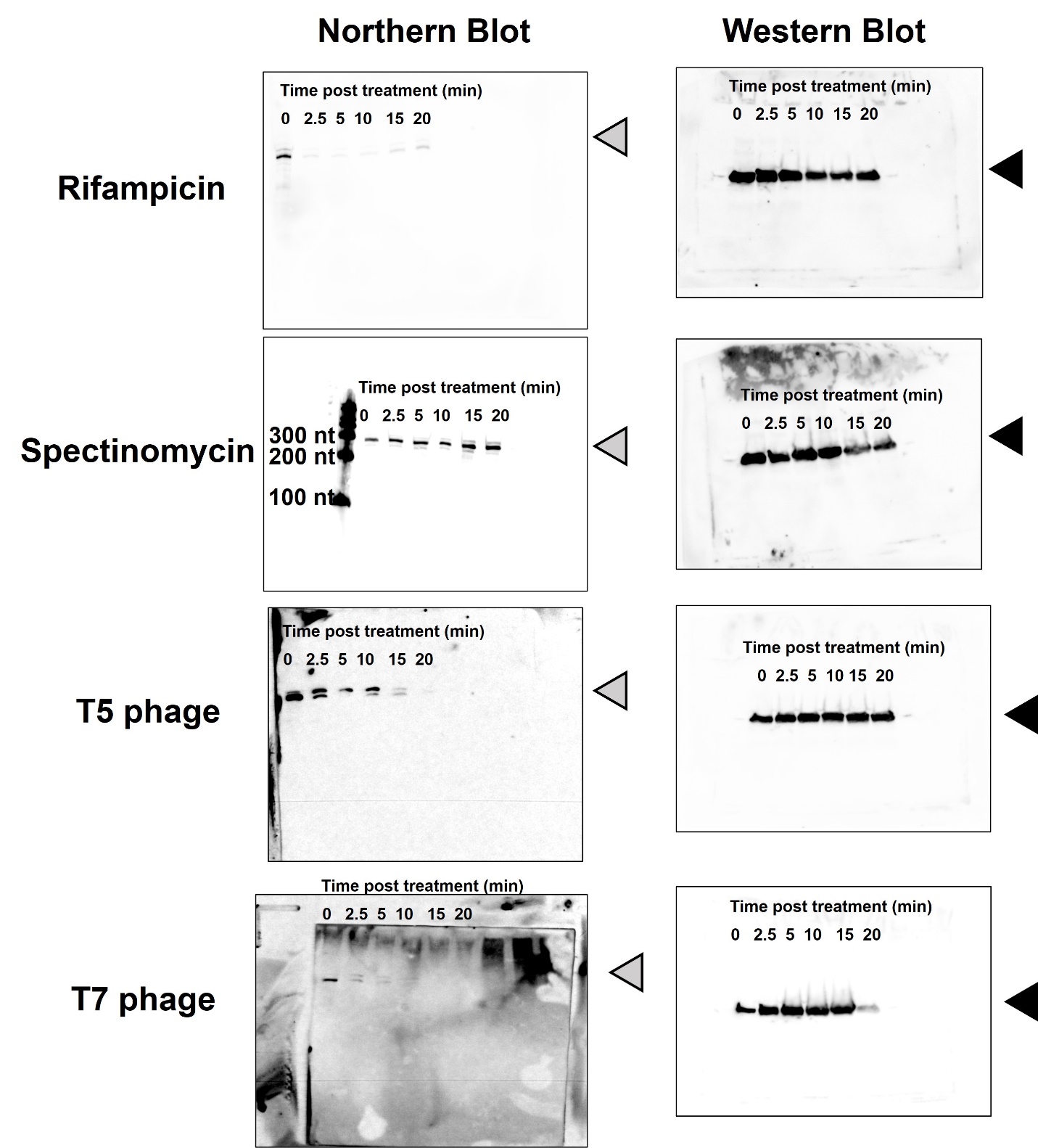
**

**S1 Fig. The raw images of the Northern blots and Western blots of Fig 1.** Shown are the raw images of Northern blots of *avcI* RNA and Western blots of AvcD-6xHis during rifampicin treatment, spectinomycin treatment, T5 infection, and T7 infection. Gray triangle corresponds to *avcI* transcript (~280 nt) and black triangle corresponds to AvcD-6xHis (60 kDa).
