# Supplementary material for: Replication cycle timing determines phage sensitivity to a cytidine deaminase toxin/antitoxin bacterial defense system
Source: PLoS Pathog. 2023 Sep 8;19(9):e1011195. doi: 10.1371/journal.ppat.1011195 (PMC10511110; doi:10.1371/journal.ppat.1011195)
Supplement: S2 Fig — Transmission Electron Microscopy (TEM) of Escherichia coli bacteriophage T7 (row a) and T5 (rows b and c) infecting E. coli host carrying the active avcID (row a and c) or inactive avcID (row b). Samples were negative stained with 1% (w/v) uranyl acetate. Scale bar 100 nm. (DOCX) [file ppat.1011195.s002.docx]

**
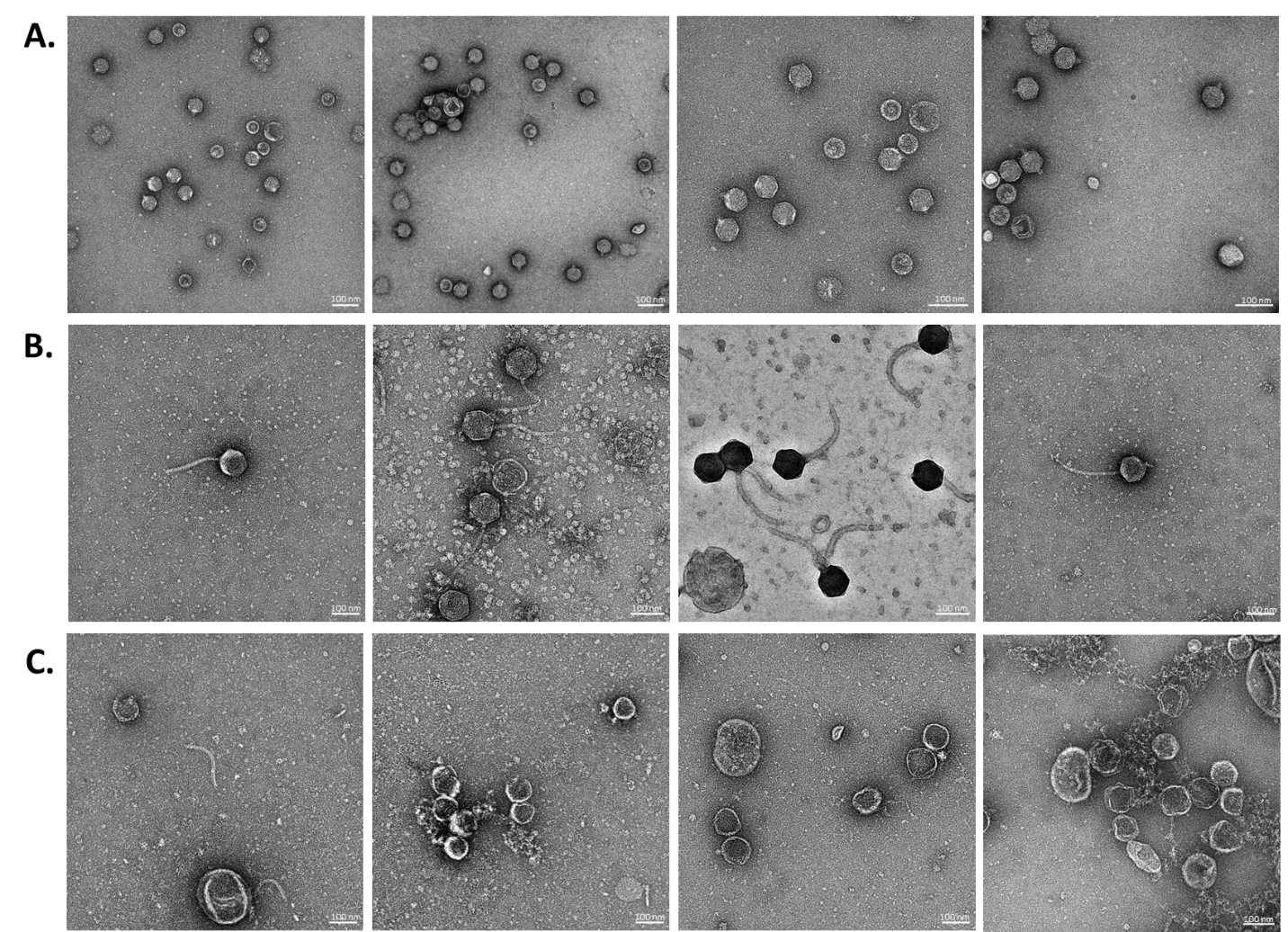
S2 Fig. Replicates of TEM Images.**

Transmission Electron Microscopy (TEM) of *Escherichia coli* bacteriophage T7 (row a) and T5 (rows b and c) infecting *E. coli* host carrying the active *avcID* (row a and c) or inactive *avcID* (row b). Samples were negative stained with 1% (w/v) uranyl acetate. Scale bar 100 nm.
