# Supplementary material for: Replication cycle timing determines phage sensitivity to a cytidine deaminase toxin/antitoxin bacterial defense system
Source: PLoS Pathog. 2023 Sep 8;19(9):e1011195. doi: 10.1371/journal.ppat.1011195 (PMC10511110; doi:10.1371/journal.ppat.1011195)
Supplement: S3 Fig — The relative genome abundance of T7 infecting E. coli MG1655 pAvcID or inactive pAvcID*. Data represent the mean ± SEM of three biological replicate cultures, two-way ANOVA with two-sided Šídák’s multiple-comparison test. (DOCX) [file ppat.1011195.s003.docx]

 **S3 Fig. AvcID shows poor protection against T7 phage infection.**

The relative genome abundance of T7 infecting *E. coli* MG1655 pAvcID or inactive pAvcID*. Data represent the mean ± SEM of three biological replicate cultures, two-way ANOVA with two-sided Šídák’s multiple-comparison test.
