# Supplementary material for: Replication cycle timing determines phage sensitivity to a cytidine deaminase toxin/antitoxin bacterial defense system
Source: PLoS Pathog. 2023 Sep 8;19(9):e1011195. doi: 10.1371/journal.ppat.1011195 (PMC10511110; doi:10.1371/journal.ppat.1011195)
Supplement: S4 Fig — Growth curves for E. coli with active (pAvcID) or inactive (pAvcID*) AvcID system after infection with untreated T7 (A, C), or MMS-treated T7 phage (B, D) at multiplicities of infection (MOI) of 0.1 or 0.0001. Data represents the mean ± SEM of three biological replicate cultures. (DOCX) [file ppat.1011195.s004.docx]

**
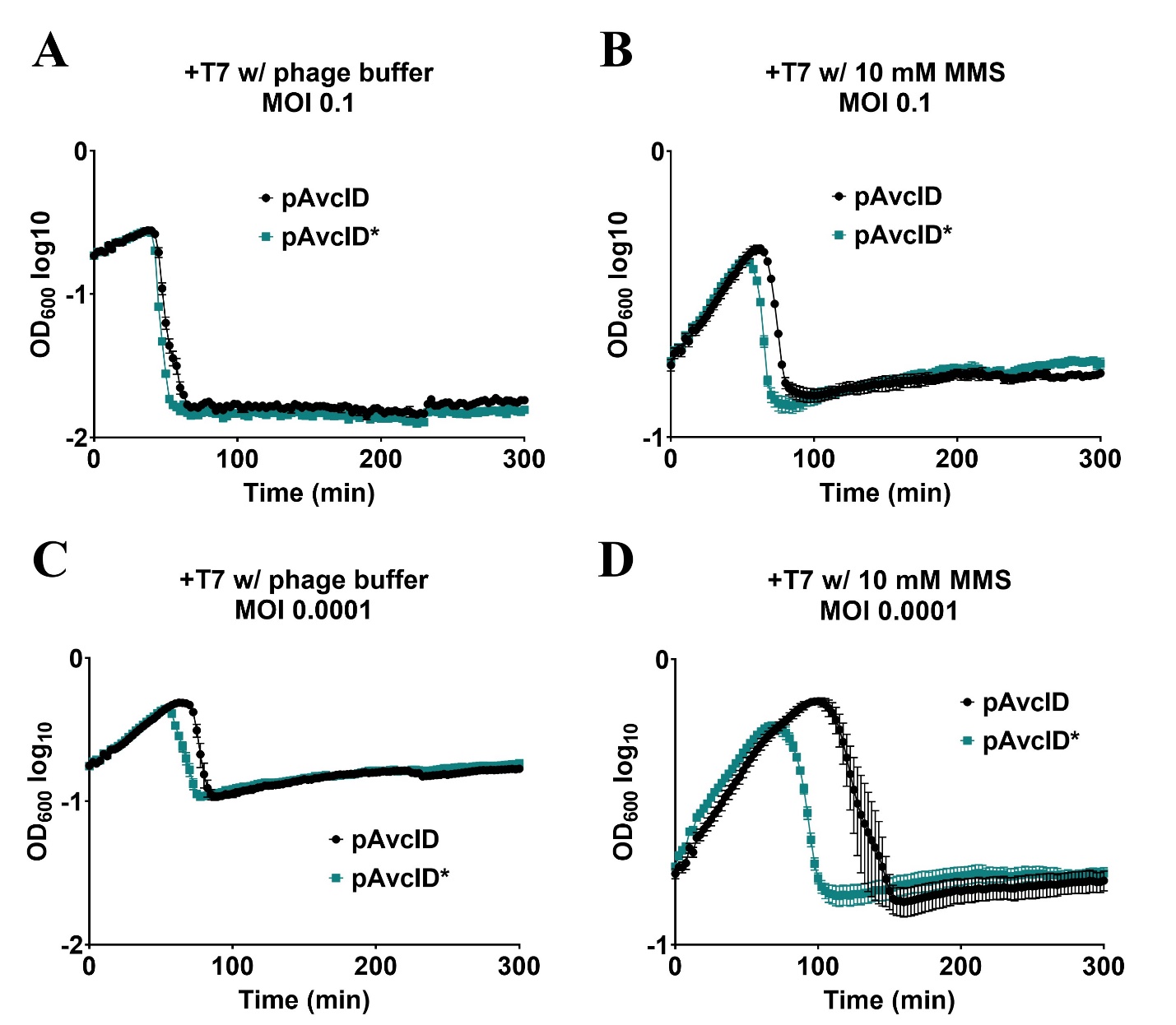
**

**S4 Fig. The AvcID system provides modest protection in *E. coli* against MMS-treated T7 phage.**

Growth curves for *E. coli* with active (pAvcID) or inactive (pAvcID*) AvcID system after infection with untreated T7 (**A, C**), or MMS-treated T7 phage (**B, D**) at multiplicities of infection (MOI) of 0.1 or 0.0001. Data represents the mean ± SEM of three biological replicate cultures.
