# Supplementary material for: Replication cycle timing determines phage sensitivity to a cytidine deaminase toxin/antitoxin bacterial defense system
Source: PLoS Pathog. 2023 Sep 8;19(9):e1011195. doi: 10.1371/journal.ppat.1011195 (PMC10511110; doi:10.1371/journal.ppat.1011195)
Supplement: S5 Fig — Growth curves of E. coli with active (pAvcID) or inactive (pAvcID*) AvcID system after infection with T7 mutants, T7C74 (A) or T7412 (B) at various MOIs. Data represents the mean ± SEM of three biological replicate cultures. (DOCX) [file ppat.1011195.s005.docx]

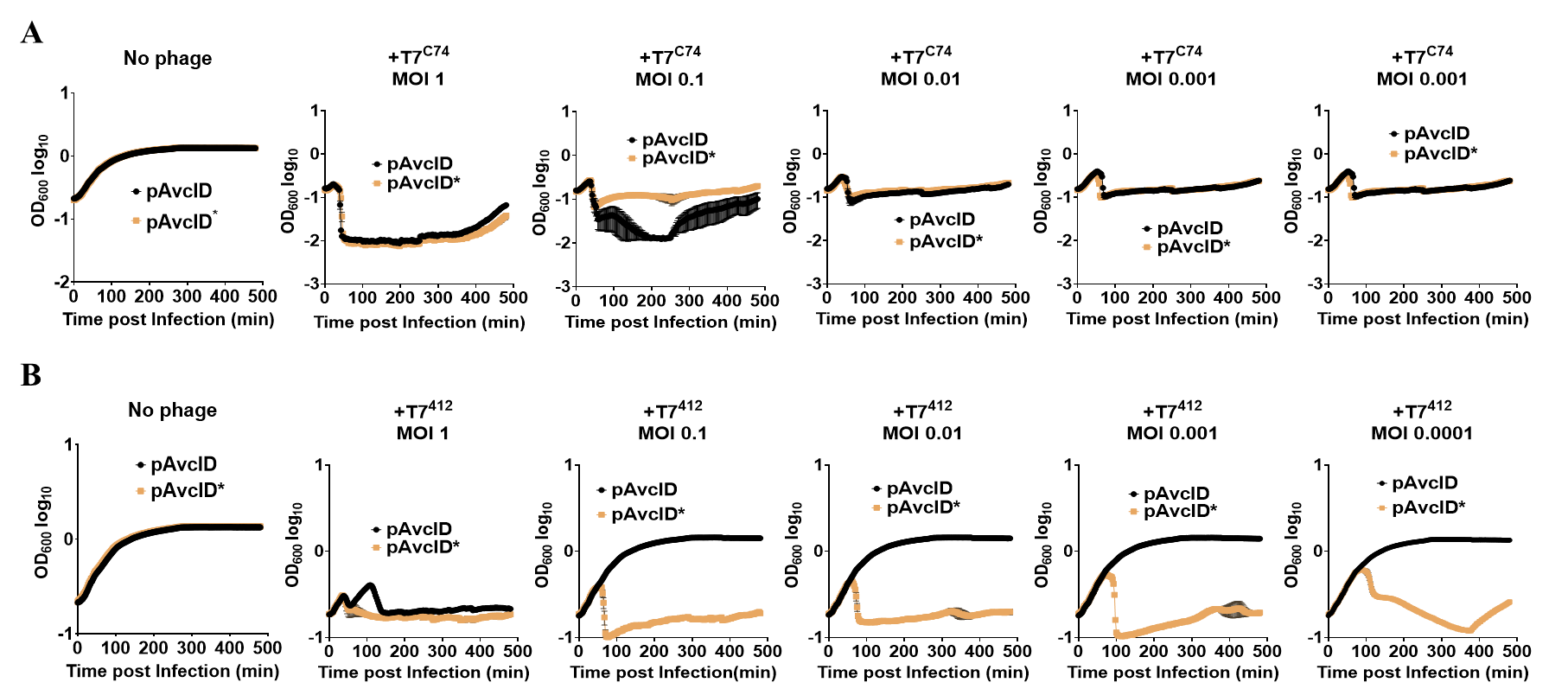


**S5 Fig. Growth curves of *E. coli* infected with T7 mutant phages.**

Growth curves of *E. coli* with active (pAvcID) or inactive (pAvcID*) AvcID system after infection with T7 mutants, T7^C74^ (A) or T7^412^ (B) at various MOIs. Data represents the mean ± SEM of three biological replicate cultures.
