# Supplementary material for: Replication cycle timing determines phage sensitivity to a cytidine deaminase toxin/antitoxin bacterial defense system
Source: PLoS Pathog. 2023 Sep 8;19(9):e1011195. doi: 10.1371/journal.ppat.1011195 (PMC10511110; doi:10.1371/journal.ppat.1011195)
Supplement: S1 Table — (DOCX) [file ppat.1011195.s006.docx]

**S1 Table.** Bacterial strains and phages used in this study.

| Strains | Name in this Study | Relevant Characteristics | Source or reference |
| --- | --- | --- | --- |
| ***E. coli*** |  |  |  |
| DH10b |  | *F-mcrA Δ(mrr-hsdRMS-mcrBC) Φ80lacZΔM15 ΔlacX74 recA1 endA1 araD139Δ(ara, leu)7697 galU galK λrpsL nupG* | ThermoFisher Scientific |
| BW29427 |  | *RP4-2(TetSkan1360::FRT)*, *thrB1004*, *lacZ58*(M15), *ΔdapA1341::[erm pir^+^]*, *rpsL*(strR), *thi-*, *hsdS-*, *pro-* | Lab Stock |
| NR8052 |  | *Δ(pro-lac) thi ara trpE9777 ung-1* | Gift from K. Yu |
| MG1655 |  | *F- lambda- ilvG- rfb-50 rph-1* | Lab Stock |
| BL21 |  | *F– ompT gal dcm lon hsdSB(rB– mB–) [malB+]K-12(λS)* | Gold Biotech |
| BL21(DE3) |  | *F– ompT gal dcm lon hsdSB(rB–mB–) λ(DE3 [lacI lacUV5-T7p07 ind1 sam7 nin5]) [malB+]K-12(λS)* | Lab Stock |
| **Phages** |  |  |  |
| T2 | T2 | Wild type | ATCC |
| T3 | T3 | Wild type | ATCC |
| T4 | T4 | Wild type | ATCC |
| T5 | T5 | Wild type | ATCC |
| T6 | T6 | Wild type | ATCC |
| T7 | T7 | Wild type | ATCC |
| λ_virulent_ | λ_vir_ | Wild type | Gift from M. Laub |
| SECϕ17 | SECϕ17 | Wild type | Gift from M. Laub |
| SECϕ18 | SECϕ18 | Wild type | Gift from M. Laub |
| SECϕ27 | SECϕ27 | Wild type | Gift from M. Laub |
| T7 mutant 412 | T7^412^ | T7 mutant; deletion of genes 0.5-1, with a non-recombining gene 1 inserted downstream of gene 12 | Gift from I. Molineux |
| T7 Mutant C74 | T7^C74^ | T7 mutant; deletion of genes 0.5-0.7 | Gift from I. Molineux |
