# Supplementary material for: Replication cycle timing determines phage sensitivity to a cytidine deaminase toxin/antitoxin bacterial defense system
Source: PLoS Pathog. 2023 Sep 8;19(9):e1011195. doi: 10.1371/journal.ppat.1011195 (PMC10511110; doi:10.1371/journal.ppat.1011195)
Supplement: S2 Table — (DOCX) [file ppat.1011195.s007.docx]

**S2 Table.** Plasmids Descriptions

| **Plasmids** | **Name in this Manuscript** | **Relevant characteristics** | **Source or Reference** |
| --- | --- | --- | --- |
| pEVS141 | pEV | pEVS143 without pTac; Km^r^ | [1] |
| pEVS143 |  | Broad-host range pTac overexpression vector; Km^r^ | [1] |
| pBYH91 | pDut | pEVS143::*dut*; Km^r^ | This study |
| pBYH67 | pAvcID | pBRP15:: *avcI-avcD* operon with its upstream intergenic region position in *V. parahaemolyticus* O1:Kuk str. FDA_R31 (CP006004) [468,152-466,174]; Amp^r^ | [2] |
| pBYH83 | pAvcID* | pBYH67::*avcD*-S47K+E376K; Amp^r^ | [2] |
| pBYH84 |  | pBYH67::*avcD*-6xHis; Amp^R^ | This study |

1. Bose JL, Rosenberg CS, Stabb E V. Effects of luxCDABEG induction in *Vibrio fischeri*: Enhancement of symbiotic colonization and conditional attenuation of growth in culture. Arch Microbiol. 2008;190:169–83.

2. Hsueh BY, Severin GB, Elg CA, Waldron EJ, Kant A, Wessel AJ, et al. Phage defence by deaminase-mediated depletion of deoxynucleotides in bacteria. Nat Microbiol. 2022;7:1210–20.
