# Supplementary material for: Replication cycle timing determines phage sensitivity to a cytidine deaminase toxin/antitoxin bacterial defense system
Source: PLoS Pathog. 2023 Sep 8;19(9):e1011195. doi: 10.1371/journal.ppat.1011195 (PMC10511110; doi:10.1371/journal.ppat.1011195)
Supplement: S3 Table — (DOCX) [file ppat.1011195.s008.docx]

**S3 Table.** Oligonucleotides Used in This Study

| **Name** | **Primer use** | **Sequence** | **Reference** |
| --- | --- | --- | --- |
| ***Vector Construction*** | | | |
| CMW3536 | *dut* F^1^ EcoRI + RBS^3^ (pEVS143-Dut) | ACAGCCTCGACAGGCCTAGGAGGAGCTAAGGAAGCTAAAATGATGAAAAAAATCGACG | This study |
| CMW3537 | *dut* R^2^ BamHI  (pEVS143-Dut) | GCTTGCTCAATCAATCACCGTTACTGACGACCAGAGTGACCAAAGCCGCC | This study |
| ***qPCR*** | | | |
| CMW3502 | *orf124_*T5 F | AGGTGCTAGCAACCACTGAC | [1] |
| CMW3503 | *orf124*_T5 R | CGTCCGATTTCGACGGTTTG | [1] |
| CMW3506 | *p52*_T7_F | CAGAACTCATGGCAAGCACG | This study |
| CMW3507 | *p52*_T7_R | TAAAGCCCTCCGCTTGGTTT | This study |
| ***Site-directed Mutagenesis*** | | | |
| CMW3456 | *avcD*-6xHis knock-in F | GTAGAAGCCACTATTGAACACCACCACCACCACCACTAACTGGCACGACAGGTTTC | This study |
| CMW3457 | *avcD*-6xHis knock-in R | GAAACCTGTCGTGCCAGTTAGTGGTGGTGGTGGTGGTGTTCAATAGTGGCTTCTAC | This study |
| ***In vitro Transcription Synthesis*** | | | |
| CMW3454 | *avcI* RNA probe F | GACCATGATTACGCCATAATACGACTCACTATAGGGTTACCAACGAATTTTCTG | This study |
| CMW3455 | *avcI* RNA probe R | [mA][mU]GGTTACAAATTTAAATG | This study |

^1^F = Forward

^2^R= Reverse

^3^RBS= Ribosomal Binding Site

1. Hsueh BY, Severin GB, Elg CA, Waldron EJ, Kant A, Wessel AJ, et al. Phage defence by deaminase-mediated depletion of deoxynucleotides in bacteria. Nat Microbiol. 2022;7:1210–20.
