# Supplementary material for: Replication cycle timing determines phage sensitivity to a cytidine deaminase toxin/antitoxin bacterial defense system
Source: PLoS Pathog. 2023 Sep 8;19(9):e1011195. doi: 10.1371/journal.ppat.1011195 (PMC10511110; doi:10.1371/journal.ppat.1011195)
Supplement: Attachment — Submitted filename: Response to reviewers.pdf [file ppat.1011195.s009.pdf]

Dear Mike,

We thank you and the reviewers for your time and excellent suggestions on this manuscript. Responding to the reviewers has significantly improved the work and strengthened our conclusions. Specifically, the most significant changes we have made are:

1. Given the confusion of Reviewer #2 with our usage of the term "lysis timing", which we were defining as the time in which a phage infects a cell to the time the cell lyses, we have now changed all such usage to "replication cycle". We hope this will clarify that we are not arguing the actual time at which the cell lyses is important for sensitivity of resistance to AvcID, but rather the speed at which a given phage undergoes its replication cycle.
2. The reviewers correctly pointed out that it is difficult to extrapolate the results we have from T5 and T7 to the other phage studied. We therefore removed the previous Fig. 8 and now focus on these two phages.
3. The reviewers correctly pointed out that the deletion of genes 0.5-0.7 in the T7-412 mutant could be responsible for increased sensitivity to AvcID. To test this idea, we performed two experiments. First, we showed that T7-412 infection of BL21(DE3), which restored early T7 RNAP expression, now resembles T7-WT and is resistant to AvcID (Fig. 7H). We also show that another T7 mutant, T7-C74, which has deletions of genes 0.5-0.7 but maintains gene 1, the T7 RNAP, in the correct location behaves like T7-WT and is resistant to AvcID (Fig. 7C, S5).
4. The reviewers wondered about the impact of AvcD activation on T7 genome replication. We thus performed a new experiment quantifying T7 genome accumulation in AvcID -/+ cells over time, and now include this as Fig. S3. This experiment showed that although there was a decrease in genome replication in the presence of AvcD, it was not statistically significant. Moreover, most phage genome replication occurs by 10 minutes. Both of these results are consistent with our conclusion that T7 replicates before AvcD is able to deplete dCTP pools.
5. The reviewers pointed out issues with the lack of delay in population decline in Fig. 1 as the MOI decreases in T5. We therefore repeated these experiments and now observe the delayed population crash as expected (new Fig. 1A).
6. We have now included more replication of the TEM analysis of defect T5 phage in Fig. S2, demonstrating that this result is robust and reproducible.
7. Due to the challenges associated with quantifying AvcI degradation, we have removed this analysis as well.

We hope that you and the reviewers find these changes significantly improve the manuscript. We would like to emphasize that there are multiple independent lines of evidence suggesting it is the speed of the replication cycle that determines resistance to AvcID. In this manuscript, we show that a delay in the replication cycle with MMS treatment or by altering when the T7 RNAP is expressed render T7 sensitive to AvcID. Importantly, an independent study by O'Hara et. al. found that decreasing the replication cycle of the *Vibrio cholerae* ICP3 phage from 40 to 20 minutes increased resistance to AvcID expressed from its native locus. This phage had only one mutation in the genome, in the phage DNA polymerase, and thus resistance is unlikely to be due to the acquisition or modification of other resistance mechanisms. Thus, we feel confident that our data and others support our model that the speed of replication cycle is a key component to AvcID resistance.

Thank you again for your time on this manuscript, and please let me know if you have any questions or if I can be of further assistance.

Sincerely,
Chris

Summary

As you will see, the two reviewers each appreciated the general topic and message of the paper, but they also each raised significant concerns. In particular, both had concerns about whether lysis timing per se is the key determinant of AvcID effectiveness, and Rev 1 raised the alternative possibility of T7 encoding an inhibitor. There were also concerns raised about some of the key technical aspects of the work, such as the MOIs used and the consequent behavior of cells infected at different MOIs. If the issues raised by the reveiwers can all be addressed, the authors should submit a significantly revised manuscript.

We cannot make any decision about publication until we have seen the revised manuscript and your response to the reviewers' comments. Your revised manuscript is also likely to be sent to reviewers for further evaluation.

Reviewer #1:

1. Figure 8: This figure is supposed to show a correlation between lysis time and the effect of the AvcID system on phage propagation. Here are several comments:

a. This is not very persuasive, as many phages with long cycle time are not effectively inhibited by the system (not only T4, but also lambda(vir) and SECphi27, both of which have a longer lysis time than T7412).

We thank the reviewer and agree with this comment. We appreciate that each phage is unique, encoding a myriad of different factors with distinct lifestyles, and therefore each should be looked at independently. In the original manuscript, we were not arguing that the replication cycle is the only factor that contributes to AvcID resistant, but one factor. However, we agree that extending our conclusions with T5 and T7 to other phages is speculative. Therefore, we have removed Fig. 8 and refocus the manuscript on how we think lysis timing is a key factor in how T7 is resistant to AvcID whereas the slower-replicating T5 is sensitive.

b. In addition, the previous work showed that this version of AvcID (from V. parahemoliticus) strongly defends against phage T3, as is also mentioned in the introduction of this paper. This is not reflected for some reason in the current data. This difference needs to be resolved. T3 has a fairly short lysis time according to figure 8, so the ability of AvcID to defend against it also refutes the proposed mechanism of selectivity between phages.

We appreciate this difference in our results, but we often find different outcomes when measuring phage defense in efficiency of plaquing assays versus liquid culture, and we do not really understand the reason for this discrepancy. Moreover, the environments that these experiments are performed in are highly impactful on the outcomes. For example, in another line of research, we have found that the activity of the *V. cholerae* CBASS system is dramatically different based on the specific experimental parameters, leading to different phage defense specificity. Therefore, as stated in the previous response, we now focus the manuscript on T5 and T7, the similarities and differences between the two, and T3 is no longer a part of the manuscript.

2. Alternative explanations for the difference between phages. The authors mention one alternative mechanism for the difference between T7 and T5 – that T7 is utilizing the degradation of the bacterial chromosome to enrich its dC pool. I assume that this implies that T5 does not do that? Could it be that the T7 412 mutant is defective in this ability? As far as I remember, chromosomal degradation is guided by early T7 genes, which are located in the same area where T7 412 is mutated. Other alternative explanations should also be discussed.

Although T5 does degrade host DNA, but it does not degrade the host genome in the same manner as T7. For most phages, host degradation and phage DNA synthesis occur simultaneously, while for T5 these two processes happen separately. In addition, during T5 infection the host DNA is degraded to nucleosides ,which are excreted from the cell rather than being incorporated into the phage genome. (PMID: 26904381).

The reviewer does make an excellent point that the T7-412 mutant is deleted for the T7 genes 0.5-0.7 in addition to relocating gene 1, the T7 RNA polymerase, downstream of gene 12. Thus, it was possible that a factor encoded by genes 0.5-0.7 is responsible for defense against AvcID. We performed two new experiments to test this idea. First, we complemented the delayed expression of gene 1 in T7-412 by infecting a BL21-DE3 mutant that expresses T7 RNAP from the bacterial chromosome. In this case, T7-412 was completely resistant to AvcID, even though genes 05.-0.7 were still missing (Fig. 7H). Second, we performed infection experiments with another T7 mutant, C74, that is also deleted for genes 0.5-0.7, but gene 1 is encoded in its normal location. The lysis timing of T7-C74 is also similar to WT T7. In support of our model, T7-C74 is resistant to AvcID (Fig. 7C, S5). These two experiments support that it is not the genes 0.5-0.7 that are responsible for phage defense, but rather the duration of the replication cyclce.

3. MMS experiment: it is very hard to understand the exact logic of the experiment. Why exactly did the authors used two different MOIs and why is the time delay between the strains with and without the system not the same in the two MOIs? As the time delay depends only on the sensitivity to defense of the first round of replication, I would assume it should be the same irrespective of the number of following rounds, no?

We used MMS treatment as previous studies have demonstrated that this delays lysis timing (Ref 31 in the manuscript). Therefore, it is another way to test our model that the speed of the replication cycle impacts resistance to AvcID. However, a delay in the replication cycle only occurs for the first round of replication, and once sufficient phages have been repaired, then the infection follows WT kinetics.

In response to the reviewer's questions, this is how we interpret the results of the MMS experiments. In the experiments in Fig. S4, we are measuring culture OD600. It is important to realize that a crash in the OD600 only occurs when the number of phages overwhelm the number of susceptible hosts. The other important point to consider is that MMS treatment of a large population of phage is stochastic, and a distribution of genetic lesions will occur such that some phage are heavily modified while others are lightly modified. At the higher MOI of 0.1, there are sufficient lightly modified phage such that the delay in population crash caused by AvcID is small (although highly reproducible). At the lower MOI of 0.001, even though a similar proportion of phage are lightly modified by MMS, additional rounds of phage replication are required for the phage to reach numbers that overwhelm the bacterial population. Thus, the delay of AvcID is more pronounced.

4. The discussion of the Dut and Ung results is a bit off the main line of argument of this paper. I

understand that the authors want to share thise piece of data and do not want to make a whole paper out of it, so they add it here. Yet, maybe they could improve the way they introduce it to the narrative of this work.

We appreciate that the Dut and Ung results are “negative” data, but we respectively disagree with the reviewer for a couple of reasons. First, these factors were actually the first model we considered that might lead to various phage susceptibility, but our data did not support this model. We feel it is important to include such testing of alternate hypotheses. Second, when presenting this work, classical bacterial physiologists often ask about the role of Dut and Ung in AvcID-mediated protection, and thus we feel it is important to show our data suggesting that these factors are not involved. We have reworded this section to try and clarify our logic here.

Reviewer #2: Major critiques:
1. Based in Fig. 1, the AvcID system only defends against phage T5 at very low MOIs (MOI≤0.001). When higher MOIs are tested, E. coli cultures expressing the active avcID system still collapse upon phage infection. Further, there does not seem to be a difference in the timing of culture collapse between different MOIs of 1, 0.1, and 0.01. The authors should: (a) plot each of the graphs in Fig. 1 on the same y axis to aid interpretation, (b) explain why the culture collapse kinetics are not different by 10-fold between MOIs of 1, 0.1, and 0.01.

We agree with the reviewer that it was odd the original data presented in Fig. 1 did not show a delay in culture collapse as the MOI decreased. We therefore repeated this experiment multiple times for T5, and the new results showed such a delay as expected. This is now shown in Fig. 1A. We also now observe complete rescue of the population at a MOI of 0.1, which we consider robust protection in a liquid environment. We have also plotted all the OD600 values on the same y-axis as suggested.

2. In Fig. 2, the authors convincingly show that inhibition of transcription leads to decreased AvcI without affecting levels of AvcD. They further show that infection with phage T5 and T7 also leads to a decrease in AvcI. However, the conclusion that T5 and T7 are decreasing AvcI via host transcription shutoff (line 180 and elsewhere) is not fully justified. Although host shutoff has been reported for both phages, these phage also rapidly destroy the host chromosome during initial steps of infection. (a) The text should be clarified to accommodate that changes in avcI levels are not necessarily the result of RNA polymerase inhibition or Southern blot analysis should be used to show the avcI is still present. (b) It is essential that the authors include loading controls (e.g. house keeping genes) for their northern and western blots in this figure to aid interpretation.

In the original manuscript, our reference to host cell transcription shutoff was meant to encompass both specific and non-specific mechanisms, like degradation of host cell DNA. We now specifically clarify this in the text. We appreciate that loading controls are critical when making quantitative measurements of Western or Northern blots, but as we no longer perform such quantification and describe these results qualitatively in the manuscript, we argue that such loading controls are unnecessary. All blots were repeated multiple times and yielded the same conclusions.

3. Figure 3 presents a central issue with the manuscript. If AvcID results in a decrease in CTP/CMP in phage T7 similar to the decrease in phage T5, why is there no phage defense? I can envision two hypotheses: either genome replication has occurred prior to 15 minutes when nucleotides are measured

or T7 genome replication is unaffected by decreased CTP/CMP. These data do not support a role of lysis timing as lysis has not been initiated but AvcID is clearly active. Yes, T7412 may argue that extending lysis timing may enhance susceptibility to AvcID, but T7412 also has numerous other issues with gene expression that could result in decreased inhibitor expression or changes in other aspects of phage physiology (as evidenced by the smaller plaques in Fig. 7). To substantiate their current conclusions, the authors should clarify how T7 lysis timing, which is still after AvcID activation, results in no decrease in PFU production. Alternatively, T7 genome replication and CTP/CMP should be measured at earlier timepoints. These experiments may reveal that it is not lysis timing, but rather genome replication timing that is the important driver in AvcID susceptibility.

We appreciate these excellent points by the reviewer. As discussed in response to reviewer 1, we have performed two additional experiments that support it is the delayed expression of gene 1, and not the loss of genes 0.5-0.7, that lead to AvcID susceptibility.

We have also now quantified genome replication of T7 upon infection of AvcID and AvcID* cells using Q-PCR, and this is now shown in Fig. S3. Importantly, most genome replication had occurred by 10 minutes, consistent with our model that such replication occurs before AvcD can effectively reduce dCTP concentrations. Although there was decreased phage genomes in AvcID infected cells versus AvcID* infected cells, this difference was not statistically significant. As we argue in the manuscript and is known for T7, phage replicate significantly more copies of genomes than are packaged in capsid heads. Thus, we think any potential decrease of T7 genome replication that may be caused by AvcID is not sufficient to decrease the number of pfus generated. We previously showed using a similar experiment that the number of T5 genomes was significantly reduced in the presence of AvcID (Ref 23 in the manuscript). I am copying it here for easy comparison. Thus, these experiments are consistent with our model that T7 is able to replicate its genomes to sufficient numbers before AvcID is active whereas T5 is not.

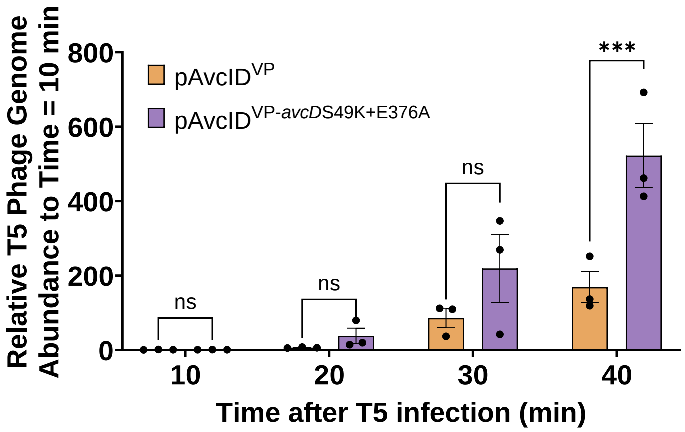

The reviewer makes a good point that it is most likely the timing at which genomes are replicated that impacts sensitivity to AvcID, and we do not disagree. The issue here is a semantics one. In the manuscript, we use the term “lysis timing” to refer to the time at which a phage infect the cells to when it lyses it including all the processes involved therein. We are not making the argument that the specific mechanism of cell lysis impacts AvcID specificity, but we understand how our manuscript could be interpreted in this manner. We therefore have replaced the use of “lysis timing” with “replication cycle” in the revised manuscript, and we clearly define replication cycle upon its first usage.

4. For Figure 8, why is there no ΔAUC for phage T3? This phage was among those which was inhibited in a previous report (line 129). Do these findings suggest that a liquid assay is indeed not more robust than a double agar overlay assay (line 137)?

We have now removed Fig. 8 from the manuscript and focus on T5 and T7 as described in our response to Reviewer #1.

5. The characterization of “defective” phages should be improved and clarified. TEM images of defective phages are convincing (and interesting!) but inherently qualitative. Can the authors improve their analysis through quantitative analysis of TEM images?

The question of why T5 are defective after infection of AvcID containing cells is an interesting one that we do not currently understand. The evidence we have from this manuscript is the significant drop of functional phage relative to phage genomes shown in Fig. 4G, and the TEM images in Fig. 5. We are currently working to understand the nature of these defects, but fully describing this here would be outside the scope of this manuscript. To bolster our results, we have now included several other images of the phage imaged in Fig. 5 in supplemental Fig. S2 to illustrate that our observations are robust and reproducible.

Part III – Minor Issues: Editorial and Data Presentation Modifications
Please use this section for editorial suggestions as well as relatively minor modifications of existing data that would enhance clarity.

Reviewer #1: 5. CFU measurements of T5 (paragraph starting on line 185, Fig. 4A): The protocol here is unclear. Was infection done at low MOI? In this case the data in Fig. 4A should mean that with the system a collapse of the population is still observed, but is delayed by ~60 minutes compared to without the system (or with a mutated one). This goes in line with the partial effect of the system on T5 propagation that is seen by other means in this section. I think that saying that population collapse is delayed is more accurate than simply saying that there is a big difference at the one time point where there is a big difference (i.e 2 hrs, line 191).

We agree with the reviewer that in this experiment, we observed a delay in population decline with AvcID. We have altered the text to be more specific.

6. dC pool depletion (line 347): from the experiment using the Ung deletion, the authors suggest that the problem imposed by AvcD to the phage is the depletion of the dC pool and not the increase of the dU pool. One way to study this is to add to the medium dC (dCMP or dCTP) and monitor whether this rescues T5 infection. This may not work if export of this molecule is not sufficiently high, but worth a try.

We thank the reviewer for this idea, but considering most nucleotides are unable to cross the bacterial membranes we do feel it would be uninformative due to technical limitations.

7. Is AvcID an abortive infection system (paragraph starting in line 375, Fig. 1)? The authors discuss whether AvcID is an abortive infection system. Here are some comments on that.
a. As the overexpression of AvcD does not lead to cell death, we would not really expect a priory that its release from AvcI would lead to this effect. In this respect, the system is not abortive simply because the "toxin" is not toxic.
b. I think that the results shown in Fig. 1 cannot support or negate an "abortive" behavior, simply because it seems that AvcID only provides weak defense against T5 and does not block lysis by the phage (and actual release of functional virions). Under these conditions, it is very hard to say anything directly on the effect of the system of the cells decoupled from the effect of the phage.
c. It is worth noting that an MOI of 1 is anyway insufficient to easily determine an abortive infection effect as ~30% of the cells are not infected initially.

We agree that AvcID is not an abortive infection system and thank the reviewer for pointing out this text. We now have modified the discussion to clearly state this perspective.

8. AvcI degradation (Figure 2 and S1, S2):
a. One would assume that all the perturbations done by the authors in Fig. 2 should control the production of the AvcI RNA and not its degradation dynamics. It is therefore inappropriate to ascribe the difference in the decay time of the RNA under different conditions to changes in degradation. It may depend maybe on changes in the dynamics of expression inhibition. I don't have any issue with the results, just a bit more care is needed with their interpretation.

We agree with the reviewer that the levels of AvcI RNA over time are impacted by several factors including degradation. Therefore, we have decided to remove quantification of the Northern blots from the revised manuscript.

b. There are two bands in the northern blot of AvcI, as clearly seen in the T5 data – why is that? Are they the same? Do the authors quantify both of them or only one of them?

Yes, we frequently observe two bands in AvcI Northern blot experiments for unknown reasons. Importantly, the electrophoresis is non-denaturing, and thus this could be different folding states of the same RNA molecule or alternate AvcI inhibitors. We are currently examining the mechanism of AvcI inhibition of AvcD, which will clarify these issues, but such a topic is outside the scope of this manuscript in our opinion. We now specifically address the presence of two AvcI bands and our thoughts on what they might represent in both the results and discussion sections.

c. Quantification (in S2) is probably very rough. It is very clear from the images in S1 and Fig 2 (note confusion in figure references in the supp. Text, line 5) that the T5 bands in time 0 are overexposed. As this leads to overestimation of the degradation time and not to underestimation, I do not think it is very important, but it should be noted in the text when discussing the degradation time.

We agree with this comment and have removed the RNA quantification.

9. Confusing choice of words. Consider rephrasing:
a. line 101 – "Similarly, type I-IV TA systems have demonstrated that one of their primary physiological roles is to limit phage infections"

Simplified to: “Similarly, other TA systems have been shown to limit phage infections [17–20]”

b. line 105 - some words missing here – “They also are highly abundant in free-living bacteria but not symbiotic, host-associated species [22], supporting that MGEs are evolutionarily beneficial and important in bacteria that are constantly challenged by phages.”

Reworded to: “TA modules also are highly abundant in free-living bacteria but not symbiotic, host-associated species [22], supporting their role as phage defense systems.”

c. Line 137: “protection in liquid culture as this would be a more robust experimental system to explore molecular mechanism.”

Changed to: “we wondered if the AvcID system could also confer protection in liquid culture as this would be a more tractable experimental system to explore the molecular mechanism of this phage defense”

Reviewer #2: Minor critiques:
1. The authors should provide an explanation for why two bands appear for avcI in their northern blot, especially as these seem inconsistent.

As discussed above for Reviewer #1, we do not know if these bands are different folding variants of AvcI or different sized RNAs. We now mention this in the text.

2. The growth curves in Fig. 1 should show OD and growth before infection, then indicate the timepoint in which phage was added (or indicate that x-axis is minutes post-infection).

We have changed the x-axis to “post infection” to indicate that phage were added at time 0.

3. Can the authors clarify why T5 phage defense data (MOI=0.1) in Fig. 1 is inconsistent with phage defense data in Fig. 6A?

We repeated the experiments in Fig. 1A as mentioned, and we now see full protection in both experiments at a MOI of 0.1.

4. In Fig. 2D, the authors state that protein stability of AvcD is not affected. However, AvcD-6xHis is degraded after 20 minutes during infection with T7. Is this significant? Is this, perhaps, the important facet of why T7 is not impacted by AvcD?

The lower AvcD-6xhis intensity at the 20 min. time point is due to the fact most of the cells are lysed, and therefore we do not think this decrease in AvcD is significant. Importantly, the quantification of T7 genome replication in Fig. S3 that we have now added to this revised manuscript supports that most genome replication has occurred by 10 min.

5. In Fig. 4, infected bacterial pellets are separated from phage lysate before quantification of CFU/mL of the bacteria and PFU/mL of the phage. Is there a way to control for phage that have adsorbed onto the bacterial culture?

This is an interesting comment by the reviewer, and it is something that we had thought about. Unfortunately, we are not sure how to easily control for such an effect. However, we would expect this factor would be similar for AvcID -/+ cells and thus should not impact our conclusions comparing the two although it might impact the total CFU count.

6. Labels above TEM images would increase clarity and ease of comprehension (Fig. 5).

We have added labels to the TEM images.

7. T5 encodes a protein that may influence the activity of Ung (PMCID: PMC8201957). A discussion of this in relation to data in Fig. 6 would be helpful and could perhaps explain the lack of phenotype.

We have now mentioned this as a potential caveat in the discussion.

8. Figure 8: These data could be improved by inclusion of replicates, error bars, etc.

We have now removed Fig. 8.
