# Supplementary material for: Replication cycle timing determines phage sensitivity to a cytidine deaminase toxin/antitoxin bacterial defense system
Source: PLoS Pathog. 2023 Sep 8;19(9):e1011195. doi: 10.1371/journal.ppat.1011195 (PMC10511110; doi:10.1371/journal.ppat.1011195)
Supplement: Attachment — Submitted filename: Response to reviewers-second revision.pdf [file ppat.1011195.s010.pdf]

Dear Mike,

We thank you and the reviewers for your positive response on this manuscript (PPATHOGENS-D-23-00255). Below is my response to the minor edits from Reviewer #1. We look forward to this manuscript being published in *mBio* and thank you very much for your time.

1. Fig. 1B – is T7 replication time so fast and burst size so high that MOI of 0.0001 sufficient to collapse it in 100 mins? If so, why don't we see an earlier collapse with higher MOIs?

RESPONSE: Yes, we think this response makes sense. Note that T7 has a replication cycle of 22 min. Furthermore, our quantification of T7 genomes in Fig. S3 indicates that ~500 genomes are replicated at 15 minutes. Thus, if we assume a burst size of 500 phage, that means at the time of the population crash for a MOI of 0.0001 (which is more like 80 minutes, not 100 minutes), then at least three phage replication cycles would have taken place. Thus, the number of phages would be amplified 500^3, or a factor of 1.25^8. As the initial MOI is a 1/10,000 phage/CFU ratio, and the bacteria would have only increased by at most a factor of 8 due to three doublings (2^3), then there is more than enough phage to completely collapse the population, even factoring in inefficiencies in genome packaging or reinfection of new hosts. Furthermore, at a MOI of 1 the population collapses at about 25 minutes, so there is a clear difference between the high and low MOI.

2. Line 193 – which MOI is used in this experiment? I guess this is a high MOI, otherwise this result contradict Fig. 1A?

RESPONSE: We used a MOI of 0.1 for T5 and 0.01 for T7. Yes, we did observe complete protection by AvcID at a MOI of 0.1 in Fig. 1A but not Fig. 4. This could be due to many reasons. First, the phage infection studies in Fig. 1A and Fig. 4 utilized different phage stocks (they were done literally in different years), which could have slight differences in phage viability of the stocks. Furthermore, the infection conditions were quite different. Fig. 1 was performed in a 96-well plate while Fig. 4 was done in a 10 mL culture in a shaken flask. Thus, we would argue that different outcomes at similar MOIs are not entirely unexpected.

3. Line 108 – typo in "deoxyctidine"

RESPONSE: Fixed, thank you for catching this.
